# Amended Dental Law of Ohio

**Published:** 1892-05

**Authors:** 


### DENTAL LAW.

### Amended Dental Law of Ohio.

SECTION 1. *Be it enacted by the General Assembly of the State of Ohio*, That sections 4404 and 6991 of the Revised Statutes ot Ohio be so amended as to read as follows:

SEC. 4404. From and after July 4, 1892, it shall be unlawful for any person to practice dentistry in this State, unless such person shall have first obtained a certificate of qualification issued by the State Board of Dental Examiners of this State, as hereinafter provided:

1. A Board of Dental Examiners, to consist of five practicing dentists, resident in this State, is hereby created, whose duty it shall be to carry out the purposes and to enforce the provisions of this act. The members of the first Board of Dental Examiners under the provisions of this act shall be appointed by the Governor of the State on or before the first day of May, 1892. The term for which members of said board shall be appointed shall be three years, and until their successors shall be duly appointed and qualified, and no person shall be appointed for or serve to exceed two terms in succession. All vacancies in said board caused by expiration of term, or otherwise, shall be filled by the appointment of the Governor of the State.

2. Said board shall have power to make reasonable rules and regulations for the purpose of carrying out and enforcing the provisions of this act. It shall choose one of its members president, and one secretary; and shall hold two regular meetings in the city of Columbus, on the last Tuesday in May and November, in each year, and at such other times as may be deemed necessary by said board. A majority of said board shall at all times constitute a quorum thereof for the transaction of business, but a less number may adjourn from time to time. The board shall keep full minutes of all of its proceedings, and a full register of all persons licensed and certified as dentists by said board, which shall be public records, and at all reasonable times open to inspection as such. A transcript of any of the entries in such minutes and register, certified by the secretary under the seal of said board, shall at all times and places be competent evidence of the facts therein stated. The members of the board shall have power to administer oaths, and the board shall have power to hear testimony in all matters relating to the duties imposed upon it by law.

3. Any and all persons who shall desire to practice dentistry in this State after July 4, 1892, except such persons as have been regularly since July 4, 1889, engaged in the practice of dentistry in this State, or who may hold, or may hereafter obtain diplomas from any reputable dental college, shall file application in writing with the Secretary of said Board of Dental Examiners for examination and license, and at the time of making such application shall pay to the secretary of said board a fee of ten dollars; and each applicant shall present himself before said board at its first regular meeting after filing his application for examination by said board. The examination shall be of an elementary and practical character, but sufficiently thorough to test the fitness of the applicant to practice dentistry. The examination may be written, or oral, or both, at the option of the board, and shall include the following subjects, to wit: anatomy, physiology, chemistry, materia medica, therapeutics, metallurgy, histology, pathology, and operative, mechanical and surgical dentistry. All persons successfully passing such examinations, or who may legally hold diplomas from any reputable college of the United States, or any foreign country, or who may have been regularly since July 4, 1889, engaged in the practice of dentistry in this State, of good moral character, shall be registered and licensed by said board of dentists, and shall receive a certificate of such registration and license duly authenticated by the seal and signature of the president and secretary of said board; and in no case shall the examination fee be refunded.

4. Every person receiving such a certificate of registration and license as dentist shall, before engaging in the practice of dentistry in this State, place and retain in place while engaged in the practice of dentistry in this State, such certificate of registration and license in a conspicuous position at his place of business, in such a manner as to be easily seen and read.

5. Every person who may legally hold a diploma from any reputable dental college in the United States, or any foreign country, or who has been regularly since July 4, 1889, engaged in the practice of dentistry in this State, shall, upon application and payment of a fee of two dollars, to the secretary of said board of dental examiners, and producing satisfactory and reasonable proof of the fact that he holds such diploma, or has been so engaged in the practice of dentistry in this State since July 4, 1889, receive a certificate of registration and license to practice dentistry in this State. Every applicant for license to practice dentistry under the provisions of this section shall, in person, by mail or otherwise, produce for the inspection of the board of dental examiners his diploma, or the affidavit of himself and two freeholders stating that he has been regularly engaged in the practice of dentistry in this State, and at what place or places, since July 4, 1889; and if the board of dental examiners shall, upon inspection thereof, find that the applicant is legally qualified under the provisions of this act to practice dentistry in this State, the secretary shall, without unnecessary delay, deliver to the applicant a certificate of registration and license to practice dentistry in this State, or forward the same without expense to the board in such manner as the applicant may direct. The certificate of the secretary of said board of dental examiners, under the seal of said board, stating that any person is a registered and licensed dentist, shall be *prima facie* evidence that such person is entitled to practice dentistry in this State.

Sec. 6991. All persons shall be said to be practicing dentistry within the meaning of this act, who shall for a fee, salary or other reward paid, or to be paid, either to himself or to another person, perform dental operations of any kind, treat diseases or lesions of human teeth or jaws, or attempt to correct mal-positions thereof. But nothing contained in this act shall be taken to apply to acts of *bona fide* students of dentistry done in the pursuit of clinical advantages under the direct supervision of a preceptor who is a licensed dentist in this State, or while in attendance upon a regular course of study in a reputable dental college, or to the acts of legally qualified physicians and surgeons.

1. Out of the funds coming into the possession of the board as above specified, the members of said board may each receive a compensation in the sum of five dollars for each day actually engaged in the duties of their office as such examiners; and a mileage of three cents per mile for all distance necessarily traveled in going to and coming from the meetings of the board. Said expenses shall be paid from the fees and assessments received by the board under the provisions of this act, and no part of the salary or other expenses of the board shall ever be paid out of the State treasury. All moneys received in excess of the said per diem allowance and mileage as above provided for, shall be held by the secretary of said board as a special fund for other expenses of said board and carrying out provisions of this act, he giving such bond as the board shall from time to time direct.

2. Any person who shall violate any of the provisions of this act, shall be guilty of a misdemeanor, and upon conviction thereof may be fined not less than twenty-five dollars nor more than one hundred dollars, or be confined not less than ten days nor more than one month in the county jail, or both. All fines thus received shall be paid into the common school fund of the county in which such conviction takes place. It is hereby made the duty of the prosecuting attorney of each county in the State to prosecute every case to final judgment whenever his attention shall be called to a violation of the provisions of this act.

3. Any person who shall knowingly or falsely claim or pretend to have or hold a certificate of registration, or who shall falsely and with intent to deceive the public, claim or pretend to be a registered and licensed dentist, not being such a registered or licensed dentist, shall be deemed guilty of a misdemeanor and shall be liable to the penalties provided in this act.

4. The board of examiners created by this amended act may sue or be sued, and in all actions brought by or against it, it shall be made a party under the name of the Board of Dental Examiners of the State of Ohio, and no suit shall abate by reason of any change in the membership of said board.

Sec. 2. Said original sections 4404 and 6991, to which this is amendatory, are hereby repealed.

Sec. 3. This act shall take effect and be in force from and after its passage.

